# Effect of high up front charges on access to surgery for poor patients at a public hospital in New Mexico

**DOI:** 10.1186/1475-9276-5-6

**Published:** 2006-06-23

**Authors:** Will Kaufman, Augustine S Chavez, Betty Skipper, Arthur Kaufman

**Affiliations:** 1 University of New Mexico School of Medicine, MSC08-4720, 1 University of New Mexico, Albuquerque, New Mexico, 87131, USA; 2 Department of Family and Community Medicine, MSC 09-5040, 1 University of New Mexico, Albuquerque, New Mexico, 87131-0001, USA

## Abstract

**Background:**

A public hospital in New Mexico required collection of 50% of estimated costs prior to elective surgeries for self-pay patients. This study assesses the impact of this policy on access to elective surgical procedures.

**Methods:**

Chi-square tests determined if there was a statistically significant difference between the number of self-pay and insured patient cancellations for financial reasons. A multivariate binomial regression model was used to calculate risk ratios and confidence limits for effects of race/ethnicity, and insurance status, controlling for gender, on these cancellations.

**Results:**

Of the 667 cancellations, there were 99 self-pay and 568 insured patients. Cancellations for financial reasons occurred in 55.6% of self-pay and 9.3% of insured patients (p < 0.0001). Inability to pay 50% up front accounted for 76.4% of self-pay patient cancellations for financial reasons. Self-pay, non-Hispanic whites and minority race/ethnicities were 8.76 and 8.61 times more likely to cancel for financial reasons, respectively, than insured non-Hispanic whites.

**Conclusion:**

Self-pay patients, regardless of race/ethnicity, have elective surgical procedures cancelled for financial reasons significantly more often than insured patients. The hospital's 50% up-front payment policy represents a significant financial barrier to accessing elective surgical procedures for self-pay patients.

## Background

There are approximately 45 million people without health insurance in the United States [1]. The problem of the uninsured is one of special relevance to New Mexico, which ranks 2nd in the percent of the total state population that is uninsured and 5th in the percent of children 18 years old and younger who are uninsured [2]. Ethnic and racial minorities are medically uninsured at a higher rate than the national average [3]. In addition, a 2003 Institute of Medicine review of the literature on disparities in quality and access to health care, found that even when minorities are insured at the same level as whites, they may experience barriers to accessing care due to language, geography, and cultural familiarity. This review also found that the financial and institutional arrangements of health systems along with their legal, regulatory, and policy environments may have disparate and negative effects on minorities' ability to attain quality care [4].

The majority of New Mexicans are ethnic or racial minorities, 42.1% Hispanic, 9.5% Native American and 1.9% African American [5]. The majority of uninsured both nationally and in New Mexico are working individuals and their families who do not receive employer-based coverage, do not make enough money to buy their own health insurance, and make too much money to qualify for Medicaid (a federal program operated by the states to help pay costs for the very poor and financially needy citizens). A substantial portion of the uninsured population, especially in a U.S.-Mexico Border state like New Mexico are undocumented immigrants who, legally, are not eligible for federal or state entitlement programs like Medicare or Medicaid. Fearing arrest and deportation, many in this population avoid registering at clinics for basic and preventive services, leaving them vulnerable to preventable, but serious illnesses for which they subsequently seek expensive, crisis care in emergency rooms.

Over 70 percent of uninsured Americans are in families where there is at least one full-time worker; 12 percent are in families with part-time workers; and only 19 percent are in families with no connection to the workforce [6]. Without any third-party reimbursement available to help pay for needed health care, the uninsured are often forced to pay for the entire cost. This is difficult, if not impossible for many, and carries with it financial- and health-related consequences. These consequences are compounded by the fact that the uninsured pay more for their health care at the time of service than those who are insured.

Medicare is a federal program that assists almost all elderly people and many disabled in paying their medical costs. Hospital administrators are often confused by federal Medicare regulations, and assume that they must charge all payers the same amount of money for the same services. They are unaware that they are permitted to discount services for the uninsured and for those who do not qualify for public assistance. Insurance companies and other third-party payers, like Medicaid and Medicare, negotiate discounts for their clients, while uninsured patients continue to pay the full, non-discounted rates [7]. This phenomenon is reflected in the way a public hospital in New Mexico billed "self-pay" patients, meaning those who have no third party to help pay the cost of their medical expenses. The policy stated that for self-pay patients receiving elective surgeries and admissions, "...it is the policy . . . to collect 50% of estimated charges at time of service and bill patients for remaining reasonable and customary charges."

This study was undertaken to explore the impact of this 50% up-front fee policy for self-pay patients on access to medical care. The authors explored whether cancellations of elective surgical procedures were more often related to financial reasons for self-pay patients than for insured patients.

## Methods

The authors reviewed records of patients who cancelled elective surgical procedures at the public hospital between March 1 and December 31, 2003. The term "elective surgery" refers to any surgery that is not an emergency. An elective surgery can be anything from a cosmetic rhinoplasty to a cholecystecomy for chronic biliary disease, to open reduction and internal fixation of a fractured limb. These records were available at the Hospital Admissions Department in paper form. Data collection was performed in person by two of the authors (WK, ASC) in the hospital admissions office between January 12th and February 6th, 2004. The data were collected initially as an internal review for the department of hospital admissions and later analyzed by the authors once the study was approved by the University's Human Research and Review Committee.

All records listing the scheduled date of the cancelled procedure were included. The following information was recorded in a Microsoft Excel spreadsheet: race/ethnicity, age, financial code, reason for cancellation as recorded by Admission Department staff, and, when recorded, procedure being cancelled. Financial codes designate the insurance status of the patients. Reasons for cancellation were categorized as "financially related," "not financially related," and "unknown." A cancellation was categorized as "financially related" if the reason for cancellation was due to an inability to pay any upfront charge, any refusal by the patient's insurance company to approve a procedure, or a cancellation due to a patient's insurance status pending approval. All other recorded reasons for cancellation were categorized as "not financially related." The vast majority of cancellations that were not financially related were due either to patients not keeping their surgical appointment or to the patient or physician changing the schedule. All cancellations for which there was no recorded reason for cancellation were categorized as "unknown."

The amount of the down payment was calculated and recorded on cancellation records by the Admissions Department staff. In order to keep the records confidential and ensure that there were no errors in data collection, the authors assigned a unique number, unrelated to the medical record number, to each record form. The research protocol was reviewed and approved by the Human Research and Review Committee at the University of New Mexico Health Sciences Center.

Before data collection began, the estimated total number of cancellations in our sample was 700, with an estimated 100 self-pay cancellations and insured cancellations of 600. These sample sizes gave us 80% power for detecting a difference between payment groups if 33% of the self-pay patients cancel for financial reasons and 20% of the insured patients cancel for financial reasons. We used the chi-square test to determine if there was a statistically significant difference in reasons for cancellation between self-pay and insured patients. A multivariate binomial regression model was used to calculate risk ratios and confidence limits for the effects of race/ethnicity, and insurance status, controlling for gender, on cancellation for financial reasons. Calculations were done using SAS Version 8.2.

## Results

During the study period, there were 667 patient cancellations recorded. Of these, 99 (14.8%) were self-pay and 568 (85.2%) were insured patients, including patients receiving county assistance. Of the cancellations, 55.6% (n = 55) of those for self-pay patients were for financial reasons compared to 9.3% (n = 53) for insured patients (p < 0.0001) (Table 1). Of patients who cancelled for financial reasons, inability to pay the 50% "up-front" fee was the reason given for 76.4% (n = 42) of self-pay patients.

**Table 1:** Reasons for cancellation by payment category and demographic characteristics.

Variable	N	Reason for cancellation			Chi-square p-values	
		
		Non-financial	Unknown	Financial	Three Categories	Two Categories^1^
Payment category						
Insured	568	269 (47.4%)	246 (43.3%)	53 (9.3%)	<0.0001	0.02
Self-pay	99	34 (34.3%)	10 (10.1%)	55 (55.6%)		
						
Age						
<18	96	42 (43.8%)	40 (41.7%)	14 (14.6%)	0.11	0.13
18–39	202	79 (39.1%)	78 (38.6%)	45 (22.3%)		
40–64	286	142 (49.6%)	107 (37.4%)	37 (12.9%)		
≥65	83	40 (48.2%)	31 (37.4%)	12 (14.5%)		
						
Gender						
Male	345	164 (50.9%)	108 (33.5%)	50 (15.5%)	0.02	0.006
Female	322	139 (40.3%)	148 (42.9%)	58 (16.8%)		
						
Race/ethnicity						
Non-Hispanic white	198	102 (51.5%)	79 (39.9%)	17 (8.6%)	0.002	0.04
Other race/ethnicity	468	201 (43.0%)	177 (37.8%)	90 (19.2%)		

^1^Non-financial reasons compared to the combination of unknown and financial reasons.

There were a substantial number of cancellations for unknown reasons within the insured group (43.3%, n = 246) compared to the self-pay group (10.1%, n = 10). Therefore, a sub-analysis was performed which combined the unknown reasons for cancellation and the financial reasons for cancellation for each group of patients. In this analysis, 65.7% (n = 65) of self-pay patients had unknown or financial reasons for cancellation compared to 52.6% (n = 299) of insured patients. This difference remained statistically significant (p = 0.02) (Table 1).

The multivariate analysis showed that insured minority race/ethnicity patients were 2.82 times more likely to cancel elective surgeries for financial reasons than insured non-Hispanic whites (p = 0.004). However, self-pay minority race/ethnicity patients were 8.61 times more likely (p <0.0001) to cancel elective surgeries for financial reasons than insured non-Hispanic white insured patients. And self-pay non-Hispanic whites were 8.76 times (p < 0.0001) more likely to cancel than insured non-Hispanic whites (Table 2).

**Table 2:** Multivariate risk ratios and 95% confidence limits for cancelling for financial reasons. *(This table compares financial reason to non-financial reason for cancellation while controlling for gender)*

Variable	RR (95% CI)	p-value
Payment source and race/ethnicity		
Insured and Non-Hispanic white	1.00	
Insured and other race/ethnicity	2.82 (1.38, 5.77)	0.004
Self-pay and Non-Hispanic white	8.76 (4.09, 18.80)	<0.0001
Self-pay and other race/ethnicity	8.61 (4.34, 17.08)	<0.0001

## Discussion

Our findings add to the literature on barriers to health care access faced by the medically uninsured. Compared to the insured, the uninsured are less likely to have a regular source of care, more likely to delay care, and less likely to report not receiving needed care [8]. Except for cases of severe trauma, the uninsured, compared to the insured, are less likely to be admitted to the hospital after being seen in the emergency room, are less likely to undergo recommended elective procedures, and are more than twice as likely to die in the hospital. Overall, the uninsured are less healthy and have a higher relative risk of death than the insured [9].

It has been well documented that insurance companies, along with Medicare and Medicaid, negotiate with hospitals for significant discounts when paying for the services of the patients that they cover [10]. One study of all hospitals in Illinois estimated that hospital charges were discounted about 50% on average. The same study found that the average charge for self-pay inpatients at hospitals in Cook County, including the city of Chicago, was 148% of the average amount that a major insurer had negotiated for the patients it insured [11]. The practice of charging the uninsured more is exacerbated by the fact that many hospitals maximize their charges for all patients so that they can increase the amount of reimbursement from a subset of insurance companies willing to pay the higher charges. However, while most insurance companies negotiate for their enrollees to minimize any increase in reimbursement, the uninsured patients who have no agency or company to negotiate on their behalf, are expected to pay the entire increased bill [10].

In addition to having to pay more out-of-pocket for their health care, the structure of payments can also be a barrier for self-pay patients. At times, self-pay patients are asked to pay 50% of their health care costs up front. This burdens self-pay patients, who, compared to the insured, generally have lower incomes, spend a greater portion of their income on health care, and have less ability to borrow. Consequently, they find it harder to receive medical care [12].

The higher costs for care and high up-front payments required of some self-pay patients can have serious financial consequences. One study found that safety-net hospitals have increased the aggressiveness with which they seek past due monies from self-pay patients. Bills are often passed along to collection agencies, whose aggressive collection practices are blind to the reasons these patients accrued such debt [13]. Another study found that nearly half of personal bankruptcies result from health problems or large medical bills [14]. In a study of over 7,000 uninsured patients, 60% said they needed help paying for their medical care, and 46% said they owed money to the facility where they receive their care [8].

Since the collection of data for this study, the Hospital's self-pay policy has changed. Community advocacy groups and the Governor of the State expressed growing concern about the adverse impact of this policy on access to needed services by uninsured, self-pay patients. A summit was held to address barriers to care for indigent patients at the public hospital. It was attended by the Governor, the Hospital administration, the local School of Medicine leadership, community advocacy groups, and other stakeholders in the community. One outcome was that the Executive Vice President of Health Sciences, who has authority over the hospital, reversed the self-pay policy as inappropriate for a public hospital serving a large, uninsured community. Today, self-pay patients are offered an affordable, sliding scale, up-front payment rate for doctor visits, tests, medications, hospitalizations and procedures. After receiving services, the self-pay patient is billed at a 40% reduction on reasonable charges and can arrange payment over a period of time.

The findings of this study, coupled with lessons learned from the subsequent change in this public hospital's self-pay policy in response to community advocacy, raises a broader, national health policy question. Should there be an official liaison group that could negotiate with public hospitals for affordable payment rates for the uninsured (self-pay) patients aiming to achieve comparable discounts enjoyed by the insured and by those covered by Medicare and Medicaid?

This study had limitations. The elective surgical procedure cancellation records were incomplete, for the records did not include a standardized way of recording the reason for cancellation. Many records had no reason for cancellation recorded at all. The initial intended use by the admitting department of these records was to track only the occurrence of surgical procedure cancellations. The admitting department staff was under no obligation to write down the reason for cancellation on the cover of the cancellation records. This resulted in a large number of records with no reason for cancellation recorded. This made it difficult to assess accurately the magnitude of the 50% "up-front" pay policy on access to needed surgical procedures. The majority of unknown reasons for cancellation were within the insured group. Even when all unknown reasons for cancellation were included in the financial reason for cancellation category, self-pay patients still cancelled significantly more elective surgical procedures for financial reasons than did the insured (Table 1).

Another limitation arising from the use of these cancellation records was that they did not contain all pertinent information. Because we were studying the effect of a policy on financial reasons for cancellation, income of the patients would have been useful. Unfortunately, we only had permission to view confidentially the information contained on the cancellation records where income was not recorded. Future studies of this population done prospectively should include income. A final limitation was the way in which data collection was performed. The data were collected by two of the authors in person with designation of reason for cancellation category being a judgement decision of the two authors together.

Further research in this area is planned to determine the health outcomes and hospital utilization patterns of self-pay patients who did not receive elective surgery for financial reasons. Formulating a cancellation tracking form with a specific space for reason for cancellation and more strict guidelines in interpretation of category of cancellation is planned for future studies of this population.

## Conclusion

Self-pay patients at the public hospital face significant barriers to accessing needed elective surgical procedures due to the hospital's policy of requiring, in advance of admission, 50% of the estimated total fee for the procedure. This study demonstrates that regardless of race/ethnicity, self-pay patients are significantly more likely to cancel elective surgical procedures for financial reasons than are insured patients. The hospital's 50% up-front payment policy for self-pay patients represents a significant financial barrier to accessing elective surgical procedures for these patients. In addition to the evidence we found that patients cancelled needed surgeries because of this policy, it is unknown how many patients, or their physicians did not schedule needed surgeries due to knowledge of this self-pay policy. This study advances the sparse but growing body of data demonstrating the negative consequences of inequalities in pricing of medical care for self-pay patients. It helps us to understand the extent to which such pricing policies create barriers to self-pay patients seeking needed care.

## Competing interests

The author(s) declare that they have no competing interests.

## Authors' contributions

WK and ASC conceived of the study, gathered all data, and interpreted findings. BS analyzed the data and supervised all aspects of the study. AK helped to find the source of data, interpret findings and was the final editor of the text. All authors helped develop study concepts, write and review drafts of the manuscript.
